# Ocular involvement of oak processionary caterpillar hairs: Clinical outcome up to one year

**DOI:** 10.1007/s00417-024-06648-x

**Published:** 2024-11-12

**Authors:** Martin Dominik Leclaire, Friederike Elisabeth Vietmeier, Maximilian Treder, Nicole Eter, Lamis Baydoun

**Affiliations:** 1 Department of Ophthalmology, University Medical Center Muenster, Muenster, Germany; 2 ELZA Institute, Dietikon/Zurich, Switzerland

**Keywords:** Oak processionary caterpillar hairs, Hair migration, Corneal haze, Eye, Uveitis, Climate change

## Abstract

**Purpose:**

To evaluate a consecutive series of patients that presented with ocular findings after contact with the oak processionary caterpillar (OPC) during an epidemic reproduction of the OPC in Germany in 2019 and to assess the 1-year outcome of those eyes with persisting OPC hairs in the cornea.

**Methods:**

Retrospective analysis of 11 eyes (11 patients) that presented in June/July 2019 with acute ocular symptoms after outdoor activity or caterpillar nest removal. Evaluation of patients charts and slit-lamp images up to one year. Assessment of the incidence of patients with OPC-associated ocular complaints in the subsequent years (2020–2023).

**Results:**

All patients had conjunctival injection, foreign body sensation, pain, itching and/or burning sensation. In 9/11 eyes, multiple caterpillar hairs could be detected in the superficial and deeper cornea. Hair removal was attempted in 8/9 eyes; in one eye hair removal was impossible due to its deep stromal location (lost-to-follow-up). Entire hair removal was successful in 2/9 eyes, hence, six eyes with persisting hairs within the cornea were followed. Stromal haze developed adjacent to the hairs in all eyes and individual hairs disappeared (n = 4) and/or showed migration within the cornea (n = 3). Until 2023, the number of patients with OPC-associated ocular complaints decreased continuously.

**Conclusions:**

Ophthalmologists should be aware of the possibility of caterpillar hairs in patients with acute eye symptoms after outdoor acitivities, especially in early summer; the incidence may fluctuate, though. Hair removal is recommended to avoid possible intraocular migration, still residing hairs did not cause any serious long-term complications in our cohort.

**Key Messages:**

***What is known***

- Within the late spring and early summer season, mass reproduction of the oak processionary carterpillar (OPC) can lead to an increased number of patients with OPC-related eye complaints (pain, itching and burning sensation) caused by OPC hairs within a very short period of time.
- Within that season, people should be (more) actively sensitized to avoid trees with OPC hairs, take precautions and avoid eye rubing when having complaints

***What is new***

- Complete surgical removal of the hairs can be difficult and is often unsuccessful due to their tiny size and often stromal location most possibly resulting from eye rubbing
- In the longer-term, residing hairs have shown to develop haze adjacent to the hair, migrate within the cornea or disappear/resolve. Since this may go along with an inflammatory reaction, long-term follow-up and topical steroids may be useful

## Introduction

The oak processionary caterpillar (*Thaumetopoea processionea,* OPC) is a moth, which is native to southern and central Europe but its range is spreading northwards [1, 2]. It is covered with stinging fine hairs (*Setae*) measuring 100–500 µm in length and 3–7 µm in diameter. One caterpillar can bear up to 500,000 hairs that can be easily blown by the wind [1, 3].

Extraocular affections, such as skin irritations or respiratory symptoms due to the release of histamine and other kinines are common. An IgE-mediated allergic reaction, possibly leading to an anaphylactic shock, has also been reported [1, 4].

Ocular symptoms by caterpillar hairs range from conjunctival injection, corneal irritation to swelling of the eyelids [1, 5]. Four cases with ocular complications by the OPC from the south of the Netherlands have been recently reported and a Dutch national survery suggested regional associations [6].

Ocular complaints caused by caterpillar hairs may not only derive from the OPC but also from setae of different species, such as the pine processionary caterpillar, browntail moth and the garden tiger moth [7, 8]. Also, the organic material of the caterpillar hairs has been described to trigger a nodular conjunctival reaction, which has led to the term ophthalmia nodosa in such cases [9]. Still, in some studies the exact species from which the setae originated have not always been identified [10–14].

As for more severe complications, there are reports on an intraocular migration of caterpillar hairs into the anterior chamber, which induced an iridocyclitis-like anterior chamber inflammation [8, 11]. Even an intralenticular penetration of setae has been observed, [12, 15] as was the hair migration into the vitreous, possibly causing recurrent intermediate uveitis, chorioretinitis, and rarely endophthalmitis necessitating vitrectomy or enucleation [7, 13, 16–18]. In one case, a caterpillar-hair-induced-ophthalmitis resulted in an exsudative retinal detachment [14].

Since the 1990s, the areal distribution of the OPC has expanded in Germany[19]. Outbreaks in the OPC population occur [2], and in 2019, an epidemic mass reproduction was noted in some parts of Germany, attracting large media attention [20, 21]. At the Department of Ophthalmology at the University of Muenster Medical Center several patients presented within a short time interval with acute ocular symptoms after outdoor activities and probable contact with the OPC.To date, such emergencies had not occurred at our department, and the accumulation of cases posed a logistic challenge. Earlier reports mainly describe the acute symptoms and treatment, as we also did in one case of our series that has already been presented as a single case report [22]. Back then, we had no long-term follow-up available, therefore, the aim of this study was to analyze all of our cases and to highlight the long-term clinical course of eyes with residing corneal hairs, as well as the incidence of cases at our hospital in the subsequent years.

## Materials and methods

**Fig. 1:**
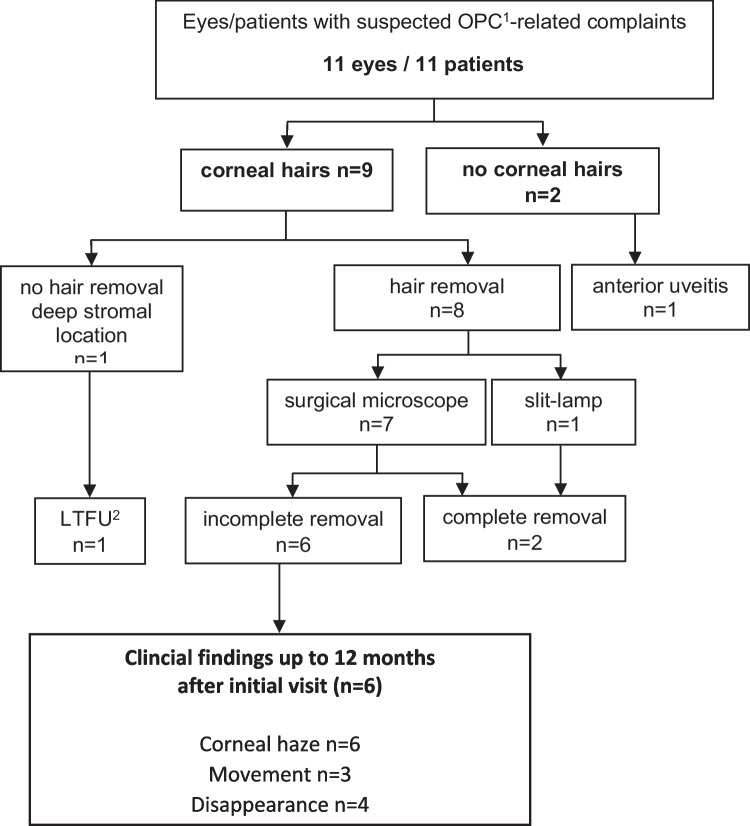
Diagram represents an overview of the study group and and the long-term follow-up

This retrospective study included 11 consecutive eyes of 11 patients with acute ocular symptoms after outdoor activity or contact with OPC hairs/nests in the early summer of 2019 **(**Fig. 1**)**. Patients´ records were reviewed retrospectively for initial symptoms, visual acuity, and complications up to 1 year.

The study adhered to the tenets of the Declaration of Helsinki. Each patient signed an informed consent form for the use of their clinical data.

All patients were examined with slit-lamp biomicroscopy, and those that presented during regular clinic times also received optical coherence tomography (OCT, Heidelberg Spectralis, Heidelberg Engineering, Germany), Scheimpflug imaging (Oculus Pentacam HR, Oculus, Germany), specular microscopy (CellChek, Konan Medical, CA, USA) and confocal microscopy (HRT RCM, Heidelberg Engineering). Best-corrected visual acuity (BCVA) was assessed and corneal OPC hairs were photographed and the patients were followed up to 1 year after the first visit.

In addition, we searched within our electronic data base file system (Fidus, Wente GmbH, Darmstadt, Germany) for further patients that presented with OPC-associated ocular complaints in the subsequent years from 2020 until 2023, using the keywords "oak processionary" and "hair".

## Results

The 11 study patients´ age ranged from 15 to 61 years with a majority of male patients (8/11 patients). All cases presented between June 23rd and July 22nd, 2019, while the first 10 cases presented within the first two weeks.

**Fig. 2:**
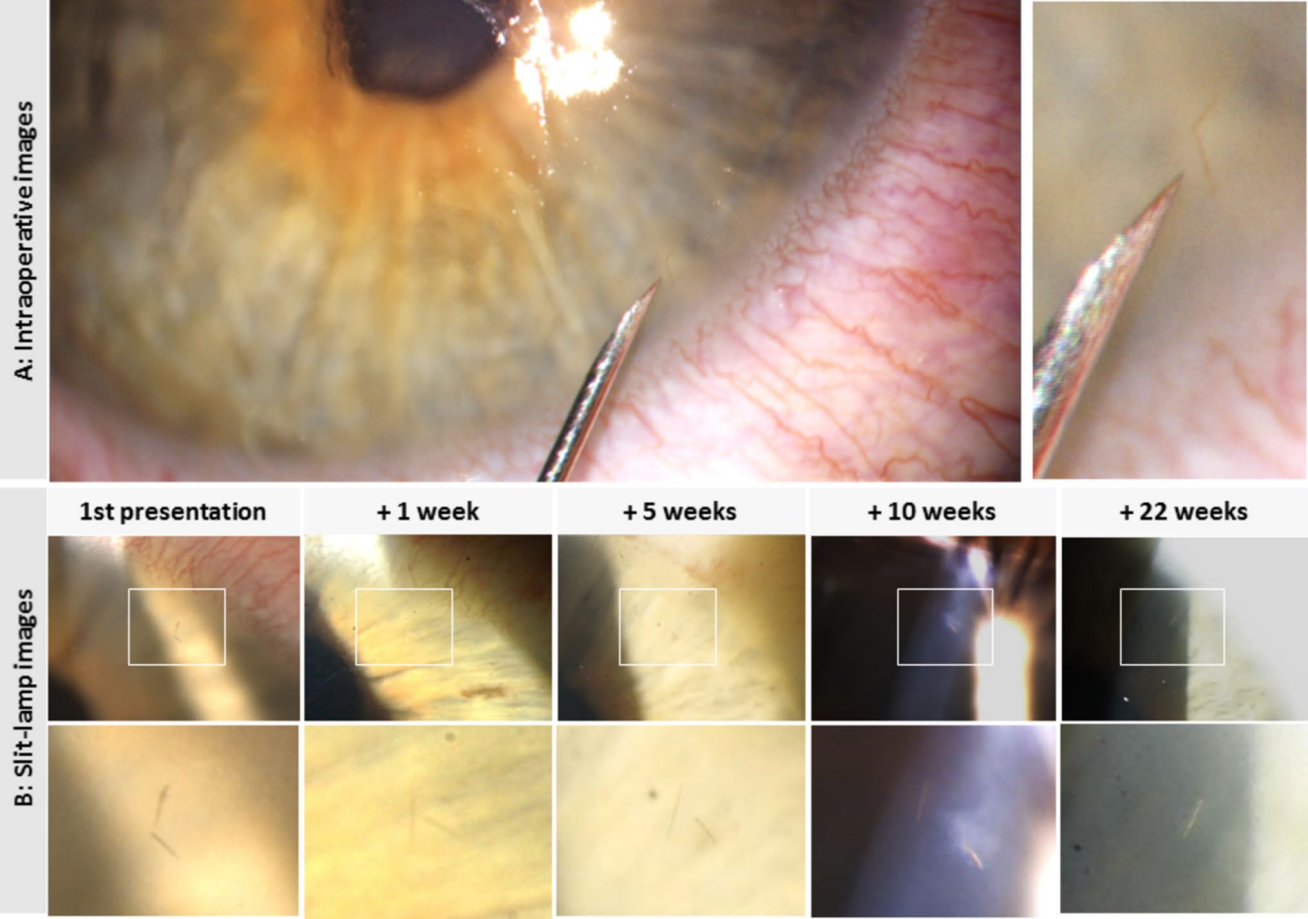
Images of corneal oak processionary caterpillar hairs at first presentation and over time of Case 6. Upper row represents pictures from the operation microscope (**A**) with an overview image of an intracorneal OPC hair next to a 30 Gauge cannula (left)and its magnification (right). Lower row (**B**) shows slit lamp images of a patient with two intracorneal OPC hairs over time with overview images (upper line) at × 10 magnification and magnified images (bottom line). Note the change in position of the hairs (setae) and emergence of a corneal haze after 10 weeks. After 22 weeks, one of the setae (that was surrounded by significant haze) has disappeared

All patients reported outdoor activities, in particular cycling or involvement (active/passive) in OCP nest removal. Patient details are displayed in Table 1, and a hair removal overview and long-term course are displayed in Fig. 1 and 2**.**

**Table 1:** Presentation of the cases with eye symptoms caused by oak processionary caterpillar hairs

Case #	First presentation	Age (Y), Gender	Ocular symptoms	Clinical findings	Outdoor activity (specification)	Number of corneal hairs at initial presentation	Hair removal Surgical microscope (SM) Slit-lamp (SL)	Complete removal	Follow-up period	Clinical findings: (H) corneal haze, (D) hair disappearance (M) hair movement
1	June 23rd 2019	57, m	Foreign body sensation	conjunctival injection	Yes (cycling)	3	Yes (SM)	Yes	n. a	n. a
2	June 23rd 2019	20, f	Foreign body sensation, pain	conjunctival injection, punctuate superficial keratitis	Yes (after opening a cabriolet roof)	1	Yes (SL)	Yes	n. a	n. a
3	June 25th 2019	15, m	Itching	conjunctival injection, swelling of eyelids	Yes (scooter driving)	8	Yes (SM)	No	12 months	H D
4	June 26th 2019	51, f	Burning sensation	conjunctival injection	Yes (cycling)	4	Yes (SM)	No	12 months	H D M
5	June 26th 2019	40, f	Burning sensation	conjunctival injection	Yes (cycling)	multiple hairs	Yes (SM)	No	2 months	H
6	June 26th 2019	28, m	Itching	conjunctival injection	Yes (cycling)	5	Yes (SM)	No	12 months^1^	H D M
7	June 27th 2019	22, m	Burning sensation	conjunctival injection, diffuse corneal edema, anterior chamber reaction with cells and two whitish tiny flakes	Yes (removal of OPC nest)	no hairs visible	n. a	n. a	n.a	n. a
8	June 30th 2019	28, m	Foreign body sensation	conjunctival injection	Yes (gardening)	5	Yes (SM)	No	3 months	H
9	July 2nd 2019	61, m	Pain	conjunctival injection	Yes (removal of OPC nest)	1	No^2^	n. a	weeks	LTFU
10	July 7th 2019	46, m	Foreign body sensation	conjunctival injection	Yes (lawn mowing)	multiple hairs	Yes (SM)	No	12 months	H D M
11	July 22nd 2019	28, m	Foreign body and burning sensation	conjunctival injection, chemosis	Yes (removal of OPC nest)	no hairs visible	n. a	n. a	n. a	n. a

^1^Suspected recurrence of conjunctival injection redness and burning sensation after discontinuation of fluorometholone eye drops 5 months after the initial visit

^2^No removal attempt due to deep stromal location of the OPC hair

*Y* years, *m* male, *f* female, *OPC* oak processionary caterpillar, *n.a*. not applicable, *LTFU *lost to follow-up

### Ocular symptoms and clinical findings

Patients that had possible contact to the OPC described foreign body sensation, pain, itching and/or burning sensation. Slit-lamp biomicroscopy revealed unilateral conjunctival injection in all eyes and three patients also had a punctuate superficial keratitis, swelling of the eyelids or chemosis, respectively. In addition, hardly visible (multiple) fine caterpillar hairs were detected in the superficial and deep cornea in nine eyes (Table 1, Fig. 1 and 2). Depending on the light exposure, the fine, partly golden brownish shining hairs were visible or invisible. The number of corneal hairs varied between a single and multiple hairs. In two patients no hairs were visible, still these are presented in Table 1 as part of the series, since they reported contact to the OPC (Fig. 1; Table 1, Cases 7 and 11). One of those, reported deterioration of uncorrected visual acuity (VA) to 1.0 logMAR (20/200 Snellen) due to diffuse corneal edema and mild anterior chamber inflammation with cells and two tiny whitish flake-like particles (Table 1, Case 7). There were no signs of vitritis or posterior uveitis and the intraocular pressure was normal. The patient did not attend further follow-up controls. Corneal edema and intraocular inflammation receded and VA recovered after topical antibiotics and dexamethasone 0.1% 6 times daily to 0.1 logMAR (20/25 Snellen) within a couple of days.

In all other 10 eyes, uncorrected VA was not or only slightly impaired (VA at initial presentation ≤ 0.2 logMAR) (Table 1).

### Imaging

OPC hairs were documented by slit-lamp photography on multiple images because one image alone could not capture all hairs due to the small size and the visibility depending on the light exposure on slit-lamp biomicroscopy.

In some of the 11 patients of our series an anterior segment OCT (n = 8) and/or Scheimpflug imaging (n = 7) and/or confocal microscopy (n = 5) was performed. The OPC hairs could not be easily visualized using these imaging modalities.

In patients in whom an endothelial cell density (ECD) measurement was obtained (n = 8), the ECD was normal at first measurement, ranging from 2680 cells/mm^2^ to 3070 cells/mm^2^. In 5 patients, follow-up ECD measurements were obtained up to 4 months to 8 months later, which showed stable results.

### Removal of caterpillar hairs

Ocular irrigation with balanced salt solution was performed in two patients. As this could not remove the hairs or reduce the hair count, further hair removal was either attempted 1. at the slit-lamp with one very superficially located hair (n = 1) or, 2. under the surgical microscope (n = 7) (Table 1). In one eye, removal of the OPC hairs was not attempted due to the deep stromal location (Table 1, Case 9). This patient was lost to follow-up two weeks after the initial presentation.

Under the surgical microscope (Fig. 2A), visualization of the hairs was also difficult despite the highest magnification, as it also depended on the angle of light that fell onto the area where the hair was located. The hairs could not be grabbed, even with the finest surgical forceps. Mobilization of the hair, by using a 30-gauge needle, was also difficult because some hairs broke and could only be partially removed while lifting them with the needle from the stroma (Fig. 2A).

Entire hair removal was successful in two eyes (n = 1; slit-lamp, n = 1; surgical microscope, Table 1, Cases 1 and 2). In six eyes, OPC hairs remained in the cornea after partial hair removal.

### Treatment and clinical course of persisting hairs

Of the six patients with persisting OPC hairs (Table 1), one was followed for 2 months (**Case 5**), one for 3 months (**Case 8**) and four for 12 months (Cases 3, 4, 6 and 10). Patients were treated with a combination of topical steroids and antibiotics for 4 weeks (dexamethasone 0.3 mg/ml and gentamicin 5 mg/ml 3 to 5 times daily) that was switched to milder steroids (fluorometholone 1 mg/ml) in the longer-term.

In all eyes, localized corneal haze adjacent to OPC hairs was noted earliest described 5 to 20 weeks after the first visit (Fig. 2B). Intracorneal movement (laterally and posteriorly towards the Descemet membrane) of OPC hairs could be observed in three eyes (Fig. 2B). Also, single hairs disappeared completely between 4 weeks to 7 months after the initial presentation.

Ocular irritation recurred in one patient (Case 6) after discontinuation of fluorometholone once a day, but subsided with topical dexamethasone.

### Review of the electronic patient file system for patients with OPC hairs 2020–2023

In the following spring/early summer of 2020, also multiple patients (although less than the year before) presented with ocular OPC hairs within a short period of time (8 patients, between 06/13/2020 and 07/13/2020). In all cases, the hairs were partially removed by corneal abrasion under the operating microscope (n = 7) or at the slit-lamp (n = 1).

**Fig. 3:**
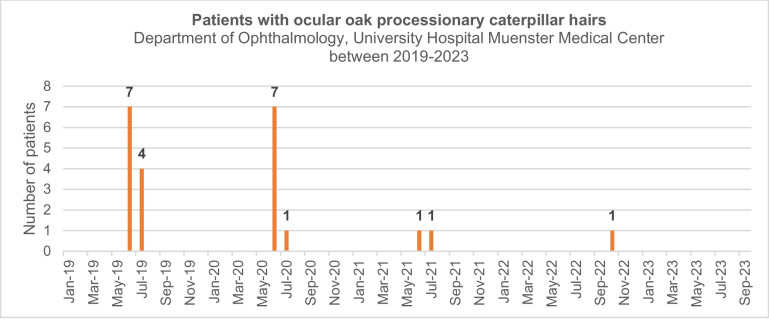
Diagram displays the number of cases that presented (as an emergency) with ocular symptoms associated with OPC hairs at the Department of Ophthalmology at Muenster University Hospital from 2019 until 2023

In the following years (Fig. 3), the number of patients further dropped. As such, in 2021 only two patients presented with corneal OPC, which were also removed by slit-lamp corneal abrasion. All patients who presented after 2019 were exclusively followed by their local ophthalmologist. None of the patients was referred to us again. In October 2022, one patient living near the German/Dutch border was referred with a severe uveitis and granulomatous endothelial precipiates presumably caused by multiple ocular OPS hairs that were located subepithelially, in the corneal stroma and near the endothelium. She had been treated with intensified prednisolone eye drops 6 times/day. Within a week, the findings improved noticeably, however, the patient did not return for further follow-ups. In 2023 no more patients presented with symptoms from ocular OPC hairs.

## Discussion

The sudden occurence of 10 cases with eye symptoms due to contact with the OPC within 14 days (11 cases within one month) may be a result of the mass reproduction of the OPC in Germany in June/July 2019 [21].

In contrast to other parts of the world [7, 15, 23], to date, ocular affections caused by OPC hairs are not common in Germany. This may change, since an increase in OPC mass reproduction may be associated with the climate change; thus the range of the OPC may expand or change [24, 25].

Affection of the skin and the respiratory system by the OPC are well known, whereas ocular affection seems to be rare and evolving in the past years [26, 27].The symptoms (e.g. itching, redness, etc.) may be misinterpreted as a conjunctivitis and an idiopathic uveitis could be suspected in the absence of OPC hairs. However, an anterior chamber reaction and even vitritis from possible intraocular hair migration have been reported earlier[6, 13]. Unfortunately, in the patient with uveitis of our series (Case 7) a causal relation with OPC hairs could not be fully proven, however the intraocular reaction with `whitish flake-like particles´ was very untypical for a common uveitis. So far, exclusively corneal extraocular location of *oak* processionary caterpillar hairs have been described [6, 28]. Notably, ocular affections of other species, like the *pine* processionary caterpillar may present differently (e.g. as conjunctival granuloma) [9]. Setae of the OPC and also of the pine processionary caterpillar may have similarities, such as microscopic spikes, which may contribute to the difficulty in removing the corneal hairs and to the similar formation of corneal haze or infiltrates, which have been observed around embedded setae of both species [6, 29, 30].In previous reports, intraocular complications caused by caterpillar hairs like vitritis, retinitis and intralenticular setae have been described but they do not provide species information and, moreover, these reports are from different regions of the world [7, 15, 23, 31]. Besides differing caterpillar species, also regional differences may affect the clinical appearance [7, 15, 23, 31]. Compared to the rather mild form of our cases, reports from the Netherlands of the same year included more severe cases [6]. Interestingly, the patient that presented with severe uveitis (in the presence of OPC hairs) in 2022 was living near the Dutch/German border.

Another striking observation was, that in all our cases, the symptoms occurred only unilaterally; bilatral affections of other caterpillar species have been observed, though [32]. Structural differences of the stinging hairs, as well as how and /where they settle may play a role when blown into the face/eye(s).

Interestingly, in the Muenster region the incidence of OPC cases appeared to decrease continuously after the primary outbreak in 2019. This could be explained by, firstly, an actual decrease in numbers of the OPC, as fluctuations of its population have been described [24]. Secondly, a rising awareness of the local population that now takes protective measures in the particular season and, thirdly, the increasing experience in the primary medical sector may have led to fewer referrals to our tertiary University Hospital.

### Visualization and documentation of (multiple) hairs

After a thorough anamnesis, a careful examination of the cornea and conjunctiva is indispensable in suspected cases. High magnification and specific light exposure was needed to visualize the hairs at the slit-lamp. At examination, we meticulously described the location of each hair in the patients´ files and photodocumented them to be able to evaluate changes over time, as a decrease in the number of hairs could indicate a possible resolution or intraocular migration. Unfortunately, multiple hairs could not be captured altogether in a single photograph. Instead, multiple pictures with high magnification were taken to acquire sharp images of most hairs seperately (Fig. 2). Documentation of the hairs by OCT, Scheimpflug imaging and confocal microsopy could not reliably identify OPC hairs or picture them easily. This may be due to a too broad distance between the single OCT scans in relation to the tiny hairs or due to the insufficient resolution of the images. Recently, caterpillar hairs were visualized by ultrasound biomicroscopy, scanning electron microscope [6, 15, 23, 31], and confocal microscopy [33]. A future prospective (multicenter) study may be useful to verify which diagnostic tool may or may not allow a consistent visualization of the hairs.

### Hair removal

Removal at the slit-lamp was possible if the hairs were spotted on the corneal surface. In the majority of our cases, however, the hairs were mainly located in the anterior and deeper stroma. This could be due to previous eye rubbing from foreign body sensation. Removal under a surgical microscope turned out to be difficult as well, since high magnification was again required, however the ocular instruments and needles were too bulky to grab the hairs. In the successive years we therefore switched to corneal abrasion of the corneal epithelium, especially when there were too many hairs. An entire removal appeared still not possible, though.

### Long-term clinical course of persisting hairs

We obserbed three phenomena despite the application of topical steroids. This included 1. intracorneal movement, 2. haze formation adjacent to the hairs, and/or 3. hair resolution/resorption (Table 1, Fig. 2).

Intracorneal movement of OPC hairs has been described earlier. Eyeball and iris movements, as well as blood pulse and respiration have been hypothesized as possible reasons [5]. Blinking, eye rubbing and the intracorneal fluid flow could be further explanations [10, 34]. The movement of hairs may also explain the disappearance of individual hairs over time, as in one of our patients one hair could eventually be spotted just anterior to the endothelium. It remains unclear whether besides lateral movement, the hairs also migrate anteriorly (extracorneally) or only posteriorly (towards the anterior chamber). In our study, except for **Case 7**, that initially showed an untypical uveitis (intraocular white flake-like particles), all other patients that presented in 2019 with corneal hairs showed no intraocular inflammation during the study period. Because of the risk of intraocular inflammation from penetration of the hairs, hair removal has also been recommended by other authors [6, 10].

Corneal haze was observed surrounding remaining setae, which may indicate a local immune reaction, which also might induce the degradation of some hairs. One of our patients (**Case 6)** reported a recurrence of the ocular irritation after discontinuation of topical steroids five months after treatment initiation. One may speculate, that residing hairs may have a persistent inflammatory potential long after the first contact. Long-term (low-potent) steroidal treatment may therefore still be required in some cases.

The retrospective design of the study is a limitation, therefore photography documentation was not available for all observed hairs as this was very time consuming, and we therefore had to rely on the description in our medical records, which have always been reported by the same examiner.

### Conclusions

This study is a long-term observation of patients with persisting corneal OPC setae. Although the clinical appearance was mild, (long-term) topical steroids may be needed in some cases to control inflammation induced by OPC hairs. Because of the risk of an intraocular migration, removal of the hairs and monitoring of the patients with persisting hairs over a longer period is recommended to initiate a timely treatment. An international registry on OPC-related symptoms, regional differences/distributions and incidences and a prospective multicenter study could provide clearer treatment recommendations and more insight to help increase awareness and improve prevention and treatment in the future.
